# Insight into the Contributions of Surface Oxygen Vacancies on the Promoted Photocatalytic Property of Nanoceria

**DOI:** 10.3390/nano11051168

**Published:** 2021-04-29

**Authors:** Yuanpei Lan, Xuewen Xia, Junqi Li, Xisong Mao, Chaoyi Chen, Deyang Ning, Zhiyao Chu, Junshan Zhang, Fengyuan Liu

**Affiliations:** 1 Department of Metallurgical Engineering, College of Materials and Metallurgy, Guizhou University, Huaxi, Guiyang 550025, China; yplan@gzu.edu.cn (Y.L.); XuewenXiaCN@outlook.com (X.X.); gz-maoxisong@outlook.com (X.M.); ndy1113@gmail.com (D.N.); ZhiyaoChuCN@outlook.com (Z.C.); JunshanZhangCN@outlook.com (J.Z.); FengyuanLiu-CN@outlook.com (F.L.); 2 Guizhou Province Key Laboratory of Metallurgical Engineering and Process Energy Saving, Guiyang 550025, China

**Keywords:** surface oxygen vacancies, nanoceria, photocatalytic property, band gap, photoinduced carriers

## Abstract

Oxygen vacancies (OVs) have critical effects on the photoelectric characterizations and photocatalytic activity of nanoceria, but the contributions of surface OVs on the promoted photocatalytic properties are not clear yet. In this work, we synthesized ceria nanopolyhedron (P-CeO_2_), ceria nanocube (C-CeO_2_) and ceria nanorod (R-CeO_2_), respectively, and annealed them at 600 °C in air, 30%, 60% or pure H_2_. After annealing, the surface OVs concentration of ceria elevates with the rising of H_2_ concentration. Photocatalytic activity of annealed ceria is promoted with the increasing of surface OVs, the methylene blue photodegradation ratio with pure hydrogen annealed of P-CeO_2_, C-CeO_2_ or R-CeO_2_ is 93.82%, 85.15% and 90.09%, respectively. Band gap of annealed ceria expands first and then tends to narrow slightly with the rising of surface OVs, while the valence band (VB) and conductive band (CB) of annealed ceria changed slightly. Both of photoluminescence spectra and photocurrent results indicate that the separation efficiency of photoinduced electron-hole pairs is significantly enhanced with the increasing of the surface OVs concentration. The notable weakened recombination of photogenerated carrier is suggested to attribute a momentous contribution on the enhanced photocatalytic activity of ceria which contains surface OVs.

## 1. Introduction

Cerium is the first element in the periodic table to possess a ground state electron in a 4f orbital (Xe 4f^1^5d^1^6s^2^), which is responsible for the powerful redox behavior between its two ionic states, Ce^4+^ (the Xe ground state) and Ce^3+^ (Xe 4f^1^) [1]. Cerium dioxide is known for its excellent redox ability, outstanding oxygen storage capacity and stable chemical properties, which make ceria a prominent function material for various applications, e.g., three-way catalysis [2], water gas shift reaction (WGS) [3,4], solar energy conversion [5,6], gas sensor [7] and chemical-mechanical polishing [8]. Meanwhile, nanoceria is also employed as one of the most attractive photocatalysts for environmental applications [9,10], clean energy generation [11,12], CO_2_ utilization [13,14,15], etc.

It is generally accepted that the photocatalytic application of ceria is impeded by its wide band gap ~3.2 eV and a quick recombination of photogenerated electrons (e^−^) and holes (h^+^) [16,17]. Attributing to the redox characteristic of Ce^4+^/Ce^3+^ pairs, oxygen vacancy is an inescapable topic for researching on ceria based catalysts [1,18,19]. Oxygen vacancies (OVs) or Ce^3+^ have been reported to affect both band structure and recombination of photocarriers significantly, and promote the photocatalytic activity of ceria [20,21,22,23]. It is believed that the OVs are favorable for reducing the e^-^/h^+^ pairs recombination rate [24,25]. Band gaps of ceria are mostly reported to be narrowed after more OVs generated [20,21,26], while a few researchers, e.g., Gao et al. [22] found the ceria with a higher OVs concentration had a blue-shift of light absorption. The existing divergent influence of OVs on ceria band gap may be interrelated to the concentration, distribution or location of OVs in ceria lattice. Though, the ceria containing more OVs shows a higher photocatalytic activity under the same light source [20,21,22,26], then the contributions of OVs on the enhanced photocatalytic property of ceria is unclear.

Reduction of the stoichiometric CeO_2_ is a main way to enrich OVs concentration in ceria lattice, including CO or H_2_ reducing [22,27,28], X-ray/UV/Ar^+^/plasma exposing [29,30,31,32,33,34] or a high temperature annealing [35]. Reducing ratio of ceria by H_2_ primarily depends on H_2_ concentration and reduction temperature, while surface oxygen may be taken away by H_2_ at a low temperature [36]. The subsurface or bulk oxygen of ceria would react with H_2_ molecules and depart away over around 850 °C, and tends to form Ce_2_O_3_ [37]. Annealing the ceria at a same temperature for the same length of time with a different concentration reductant, e.g., H_2_, is a facile way to produce ceria samples with a surface OVs concentration gradient.

Moreover, different morphologies of ceria mainly expose diverse crystal faces, where the typical morphologies including polyhedral, cubic and rod-like shape of nanoceria primarily expose the (111), (100) and (110)/(100) plane, respectively [38,39]. It is suggested that the (111) is the most stable face, and OVs are most easily formed on the (100) but which would be partially oxidized due to high surface activity, and a higher concentration of surface OVs normally exist on (110) plane [40,41,42]. The effects of OVs on the photoelectric characterizations of various shaped ceria may be different, and which still need to be systematically studied.

Thus, in present work, we synthesized polyhedral, cubic and rod-like shape of nanoceria, and annealed them at 600 °C in air, 30%, 60% or pure H_2_ to obtain ceria with various surface OVs concentration. The effects of the surface OVs on band structure, photogenerated carriers, and photocatalytic activity of different shapes of nanoceria are carefully discussed.

## 2. Materials and Methods

### 2.1. Materials

Cerium nitrate hexahydrate (Ce(NO_3_)_3_·6H_2_O, 99.95%) and sodium hydroxide (NaOH, 99.9%) were purchased from Aladdin Chemistry Co. Ltd. (Shanghai, China), which were used as received without further purification. The H_2_ and Ar gas with the purity of 99.999% were ordered from Shen-Jian Co., Guiyang, China.

### 2.2. Synthesis Process

Nanoceria was synthesized by using a simple template-free hydrothermal method under a variety of conditions to modify the morphology, which was similar to the synthetic route reported in Ref. [43]. Briefly, NaOH aqueous solutions were dropwise added into Ce(NO_3_)_3·_6H_2_O aqueous solution to form light purple mixtures with strong stirring for 30 min and then transferred to a 50 mL Teflon stainless steel autoclave, which would be maintained at a designed temperature for 24 h. More synthetic conditions are listed in Table S1. After the autoclave cooling down, all products were washed and filtered with distilled water and alcohol several times to remove impure ions, followed by drying at 60 °C in air overnight. The obtained polyhedral, cubic, and rod-like nanoceria are named as raw P-CeO_2_, C-CeO_2_ and R-CeO_2_, respectively.

### 2.3. Annealing Process

The raw P-CeO_2_, C-CeO_2_ and R-CeO_2_ were placed in a ceramic boat and then maintained in a tube furnace (gsl-1600×, Kejing, Hefei, China) for 2 h annealing at 600 °C with a heating rate of 10 °C/min under air, 30, 60 or 100% H_2_ atmosphere, respectively. Before a H_2_ annealing process, air was repeatedly expelled from the furnace tube by alternately flowing argon and vacuuming for several times. The total gas flow rate was 400 mL/min and argon was selected as the balance gas (88.79 kPa total pressure of Guiyang), and the annealed powders were henceforth named as P-CeO_2_-X, C-CeO_2_-X and R-CeO_2_-X (X = air, 30% H_2_, 60% H_2_ or H_2_).

### 2.4. Subsection Analysis

The obtained powders were subjected to several analyses. X-ray powder diffraction pattern was recorded by using X-Pert Powder (Panalytical, The Netherlands) with Cu Kα radiation (λ = 0.15418 nm) from 5.00 to 90.00° at a rate of 0.02°/s. Micrographs were taken by SEM (JSM 7610, JEOL, Tokyo, Japan) and TEM (Tecnai G2 F20, FEI, Hillsboro, OR, USA) in which the samples were ultrasonically dispersed in alcohol and dropped on the silicon wafer or copper grid. TG analysis was recorded by Mettler TGA/SDTA 851e at a heating rate of 10 °C/s from room temperature to 1000 °C with the air and 30%, 60% and pure H_2_, where Ar was used as carrier gas. H_2_-TPR was analyzed by AutoChem1 II 2920, 0.1000 ± 0.0005 g of sample was kept at 500 °C in the air for 1 h and cooled down to room temperature. After 30 min purification in Ar at room temperature, the sample was heated to 1000 °C with the mixed gas of 10% H_2_ and 90% Ar with a total gas flow of 30 mL/min and a heating rate of 10 °C/min. X-ray photoelectron spectroscopy (XPS) was recorded by K-Alpha (Thermo Fisher Scientific, Waltham, MA, USA), in which a monochromatic Al source (h*v* = 1486.6 eV) and the samples were tested in a vacuum situation of 2 × 10^−9^ mbar with C 1s peak (284.8 eV) reference. The UV-visible diffuse reflectance spectra (UV-Vis DRS) were recorded by UV 2700 (Shimadzu, Tokyo, Japan) with the wavelength from 200 to 800 nm and BaSO_4_ was used as reference, while the photoluminescence spectroscopy (PL spectra) was analyzed by FluoroMax-4 (HORIBA, France) with an excitation wavelength of 300 nm. The ultraviolet photoelectron spectroscopy (UPS) recording by ESCALAB 250Xi (Thermo Fisher, Waltham, MA, USA) was performed the valence states of all samples at the He I line (h*v* = 21.2 eV) with C 1s reference in a vacuum situation of 2 × 10^−8^ mbar. Transient photocurrent curves were recorded under a light irradiation provided by a 250 W xenon lamp in 0.1 mol/L Na_2_SO_4_ aqueous solution at bias voltage of 0.4 V, which was employed by an electrochemical workstation (CHI660C, CHI shanghai Co., Shanghai, China) in three electrode cells. The tested samples were dispersed in a nafion (10 μL), ethanol (750 μL) and deionized water (750 μL) mixture solution and further dip-coated on a glassy carbon plate (*Φ* = 3 mm), which was used as the working electrode, and a Pt plate and Ag/AgCl were employed as the counter and reference electrode, respectively. The BET surface area and N_2_ adsorption results were analyzed by ASAP2460 (Micromeritics, Norcross, GA, USA).

### 2.5. Photocatalytic Performance

The photocatalytic performances were tested by a self-built photochemical reactor, which was composed of a 250 W xenon lamp and a quartz vessel. In total, 80 mL of methylene blue (MB) solution with a concentration of 10 mg/L was used for simulating the waste dye solution, and 20 mg of synthesized catalyst was added into the reactor with ultrasound for 10 min. After 30 min of dark adsorption, the lamp was turned on and the catalyst was reacted with the MB under a light facula, 5 mL solution was sampled for 30 min each in the next 2 h. The sampled solution was centrifuged firstly with a speed of 10,000 r/min for 5 min and then the MB concentration was measured by a spectrophotometer under the maximum wavelength of 664 nm. The degradation ratio can be calculated by the following formula [44,45]:

Degradation ratio (%) = (*C*_0_ − *C_i_*)/*C*_0_ × 100%. (1)

At a low concentration of MB with a weak adsorption, the photocatalytic reaction kinetics in general follow the Langmuir–Hinshelwood (L-H), and the equation of the pseudo-first-order reaction rate constant [46] can be given as:

ln(*C_i_*) = −*kt* + ln(*C*_0_), (2)

where *C*_0_ and *C_i_* are the initial and tested concentration of MB, respectively, *k* is the pseudo-first-order reaction rate constant (min^−1^), *t* is the photocatalytic reaction time (min).

## 3. Results and Discussion

### 3.1. Phase and Morphology

**Figure 1:**
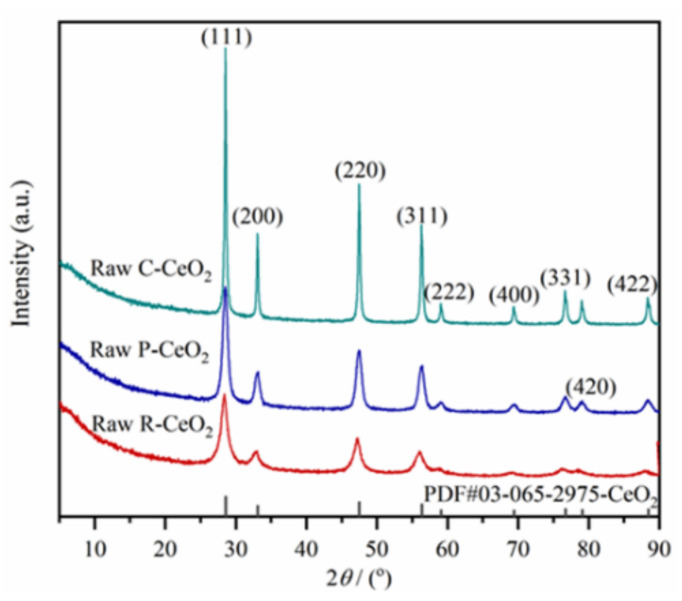
XRD patterns of raw P-CeO_2_, C-CeO_2_ and R-CeO_2._

XRD patterns of synthesized nanoceria are exhibited in Figure 1, it can be seen that all samples show the typical diffraction peaks of CeO_2_ with a fluorite-type structure and Fm$\bar{3}$m space group (PDF: #03-065-2975). The raw C-CeO^2^ has the strongest diffraction intensity, followed by raw P-CeO_2_ and R-CeO_2_, the crystal sizes of raw P-CeO_2_, raw C-CeO_2_ and raw R-CeO_2_ were calculated as 6.7, 36.5, and 10.0 nm, respectively.

**Figure 2:**
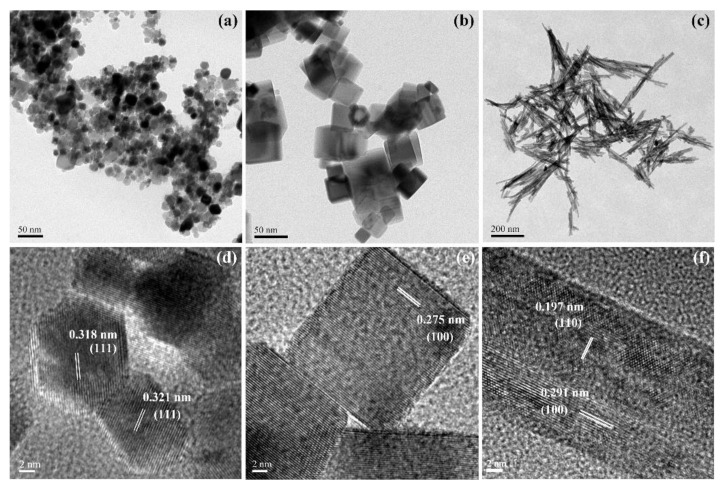
TEM images of raw P-CeO_2_ (**a**), C-CeO_2_ (**b**) and R-CeO_2_ (**c**), HRTEM images of raw P-CeO_2_ (**d**), C-CeO_2_ (**e**) and R-CeO_2_ (**f**).

In Figure 2, TEM images clearly exhibit that the synthesized nanoceria samples own the desired morphology of polyhedron, cube and rod, the counted statistical particle size of synthesized ceria is given in Figure S1, showing the average size of P-CeO_2_, C-CeO_2_ and R-CeO_2_ is around 9, 40, and 100 nm (for length), respectively. From the HRTEM images as shown in Figure 2, the spacing lattice fringes are measured as 0.318 and 0.321 nm for the P-CeO_2_ associating with presenting (111) plane, and the (100) face is found in the HRTEM image of C-CeO_2_, while both (110) and (100) planes are exposed in the R-CeO_2_, which is in agreement with the theory for the main exposing face of various shaped ceria [38].

### 3.2. Reduction of Ceria in H_2_

**Figure 3:**
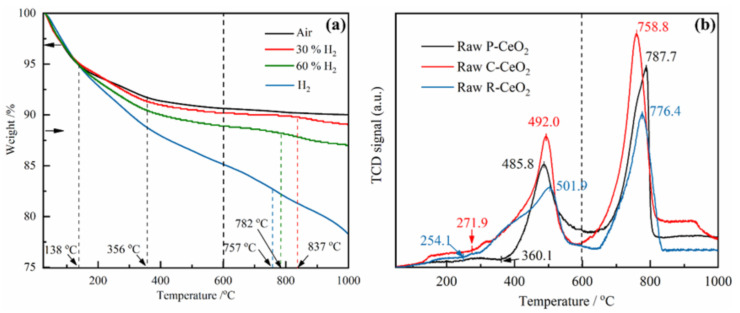
Thermal reduction behavior of synthesized ceria, (**a**) the thermo-gravimetric analysis of raw R-CeO_2_ under different atmosphere, (**b**) the H_2_-TPR of raw P-CeO_2_, C-CeO_2_ and R-CeO_2_.

The TG analysis results of raw R-CeO_2_ under different atmospheres are shown in Figure 3a, obviously weight loss can be found under each atmosphere and the weight loss increases in a higher H_2_ concentration atmosphere. In air condition, the first weight loss is about 5.6 wt. % corresponding to the vaporization of free water, then the R-CeO_2_ continues to weightlessness from 138 to 356 °C, where the weight loss is about 3.2 wt. %, which may be in connection with the Ce(OH)_4_ decomposition [47]. At a higher temperature, the weight signal of sample stabilizes at about 89.0 wt. % of initial weight. The weight loss of ceria in 30%, 60% or pure H_2_ atmosphere can be divided into four steps: (i) free water evaporation; (ii) H_2_ adsorbs on the surface of R-CeO_2_ and hydroxylates with Ce^4+^ accompanying by the water decomposing [48], which leads to around 2.9, 4.2 and 5.7 wt. % of weightlessness at 356 °C under different hydrogen concentration; (iii) the surface of R-CeO_2_ is continually and incompletely reduced by H_2_, and the decrement of weight is about 1.7 wt. % at 837 °C for 30% H_2_, 2.0 wt. % at 782 °C for 60% H_2_, and 6.0 wt. % at 757 °C for pure hydrogen atmosphere, respectively; (iv) the subsurface of ceria is reduced and tends to form Ce_2_O_3_ [37]. With the increasing of H_2_ concentration, the weight loss of raw R-CeO_2_ at the same temperature increases, which means more O atoms are divorced from ceria lattice by the following reaction:

CeO_2_ + *x* H_2_ ↔ CeO_2−*x*_ + *x*/2 H_2_O (g), (3)

where 0 < *x* < 0.5, and nonstoichiometric value of *x* depends on the temperature and H_2_ partial pressure.

The H_2_-TPR results of synthesized raw CeO_2_ are shown in Figure 3b. The first and second peak shown in TPR curves corresponds to the surface reduction and bulk reduction of ceria by hydrogen, respectively [36]. The synthesized various structural nanoceria samples show different start/end reduction temperatures for the first reduction stage of three tested samples, where R-CeO_2_ has the widest reduction range of 254.1–501.9 °C, P-CeO_2_ shows the narrowest range from 360.1 to 485.8 °C, followed by the 271.9–492.0 °C for C-CeO_2_. At the second reduction step, the maximum reduction peaks are found to be achieved at the temperature of 758.8, 776.4 and 787.7 °C for C-CeO_2_, R-CeO_2_ and P-CeO_2_, respectively. The notable diversity of the reducing behavior for the R-CeO_2_, C-CeO_2_ and P-CeO_2_ further verifies the different activities of the mainly exposed crystal faces in ceria, where (110) and (100) are more active than (111) facet [40,41,49,50]. Based on the TG and H_2_-TPR results, annealing ceria at a same temperature of 600 °C in different hydrogen partial pressure atmosphere will produce ceria samples with various concentration of surface OVs.

### 3.3. OVs Characterization

Three shaped ceria powders were annealed in four types of atmospheres, and different colored products were obtained, where the ceria annealed in air is pale yellow for P-CeO_2_ and R-CeO_2_, white for C- CeO_2_, and then turns to greyish-green or blue-yellow with the increasing of H_2_ partial pressure, and the colors of annealed ceria are shown in Figure S2. It is known that the color of pure and stoichiometric cerium dioxide is pale yellow [51], and its color will turn to blue or even black after the formation of nonstoichiometric ceria [52], the observed color variation means an abundance of OVs were generated after hydrogen annealing.

**Figure 4:**
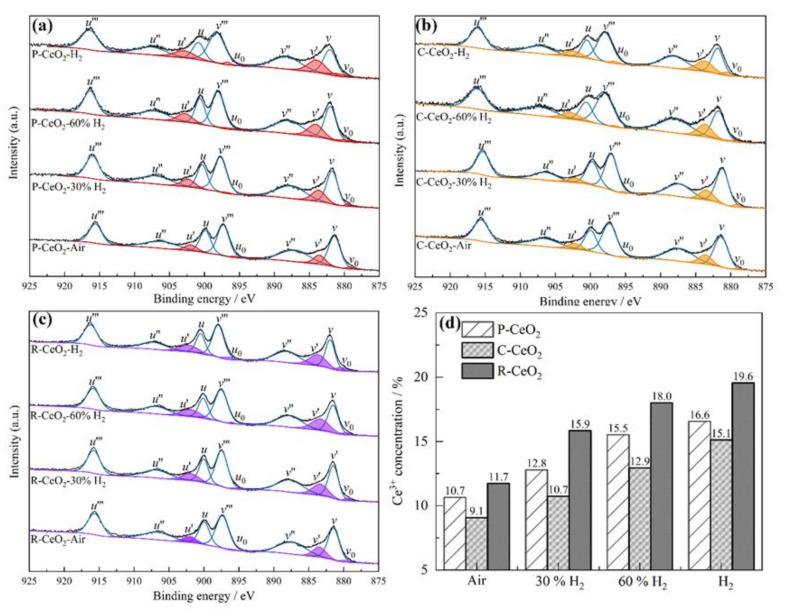
The XPS spectra of P-CeO_2_ (**a**), C-CeO_2_ (**b**) and R-CeO_2_ (**c**) annealed in different concentration of H_2_, and the calculated Ce^3+^ fractions (**d**).

The Ce 3d spectra of raw and annealed ceria are shown in Figure 4, three final states of Ce^4+^, including Ce3d^9^4f^0^O2p^6^, Ce3d^9^4f^1^Op^5^ and Ce3d^9^4f^2^O2p^4^ expressed as *u*’’’ (*v*’’’), *u*’’ (*v*’’) and *u* (*v*) for Ce3d_3/2_(Ce3d_5/2_), respectively; two final states of Ce^3+^, including Ce3d^9^4f^1^O2p^6^ and Ce3d^9^4f^2^O2p^5^, are expressed as *u*’ (*v*’) and *u*_0_ (*v*_0_) for Ce3d^3/2^(Ce3d^5/2^) [53,54,55,56]. The Ce^3+^ fraction was calculated by the following equation [57].

Ce^3+^ / (Ce^3+^ + Ce^4+^) = area(*v_0_*, *u*_0_, *v′*, *u′*) / total area. (4)

As shown in Figure 4, the areas of *v’* and *u’* corresponding to the Ce^3+^ are increasing after annealing in a higher hydrogen contained atmosphere, which means more Ce^4+^ in ceria was reduced to Ce^3+^. Calculated Ce^3+^ fractions are shown in Figure 4d, where an obvious rising of Ce^3+^ fraction can be found with the increasing of hydrogen concentration, the Ce^3+^ % increases from 10.66 to 16.56%, 9.71 to 15.12%, and 11.73 to 19.55% for the P-CeO_2_, C-CeO_2_, and R-CeO_2_, respectively. More OVs appear on the surface of R-CeO_2_ which is related to the suitable surface activity of (110) facet [58]. In O 1s spectra (are given in Figure S3), oxygen species are originated from lattice oxygen (O_L_) attached to Ce^4+^ ion and adsorbed oxygen to Ce^3+^ site (O_V_), which can be deconvoluted into two peaks at around 529.2 and 531.3 eV, respectively [59,60]. The area and intensity of O_V_ peak are relevant to the oxygen vacancy in the host lattice, which is calculated and given in Table S2. It can be found that the O_V_ fraction of ceria increases with the rising of H_2_ concentration in annealing gas, which further identified the results shown in Ce 3d spectra that more surface OVs are generated after annealing in a higher H_2_ concentration atmosphere at 600 °C.

### 3.4. Photocatalytic Activities

**Figure 5:**
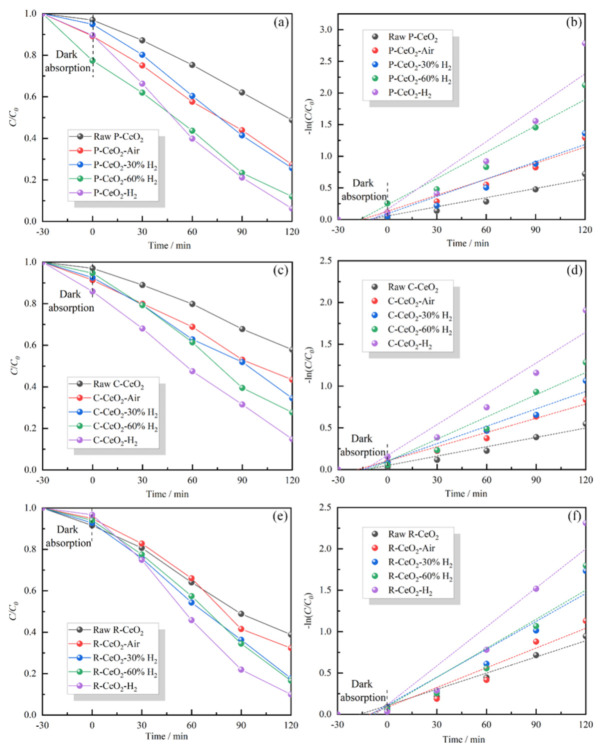
The photocatalytic degradation ratio and rate of P-CeO_2_ (**a**,**b**), C-CeO_2_ (**c**,**d**), and R-CeO_2_ (**e**,**f**) calcining in different atmosphere.

Photocatalytic properties of tested ceria are shown in Figure 5, and the photodegradation ratio of the tested samples is presented in Table S3 together with the calculated degradation rate constants. It is clearly found that the annealed ceria has a higher photocatalytic activity than that of raw material, and the photocatalytic activities of three structural ceria are elevated gradually with the increasing of surface OVs concentration. The observed results further verified the reported results [20,21,22,26] that the OVs are beneficial for the enhancement of photocatalytic property of ceria. P-CeO_2_-H_2_ has the highest photodegradation ratio of MB of 93.82%, which is larger than 90.09% of R-CeO_2_-H_2_ and 85.15% of C-CeO_2_-H_2_. The excellent photocatalytic activity of P-CeO_2_ may be due to its smallest average particle size around 9 nm.

As it is known that size [61], morphology [62] and OVs concentration [63] are the main factors which influence the photocatalytic activity as well as the photoelectric characterizations of ceria. The TEM images of the CeO_2_ annealed in air and pure hydrogen are given in Figures S4–S7. It can be seen that the annealed P-CeO_2_ still exhibits (111) facet, the crystal plane of annealed C-CeO_2_ is transformed from (100) to stable (111). The plane of calcined R-CeO_2_-Air is also tended to (111) with small particles aggregating, but the previously existing (110) facets are turned to active (100) in R-CeO_2_-H_2_ with the small holes on nanorods. The average particle size of P-CeO_2_-H_2_ and C-CeO_2_-H_2_ is larger than that of P-CeO_2_-air, and C-CeO_2_-air, respectively, but the length of R-CeO_2_-H_2_ is found as around 60 nm which is shorter than that of R-CeO_2_-air.

The XRD patterns of C-CeO_2_ annealed in air, 30%, 60% and pure H_2_ are shown in Figure S8. It can be found that all annealed samples have the similar diffraction pattern of CeO_2_, no peaks of Ce_2_O_3_ can be found. The calculated crystal size is given in Table S4, the crystal size of the ceria increased after annealing, which is in agreement with the TEM images, while the sample annealed in hydrogen has a slightly increased crystal size compared with that in air. Moreover, the nitrogen adsorption–desorption isotherms of the P-CeO_2_, C-CeO_2_, and R-CeO_2_ calcining in air or pure H_2_ are shown in Figure S9. It was calculated that the BET surface area of P-CeO_2_-air, C-CeO_2_-air and R-CeO_2_-air is 60.25, 20.94 and 68.20 m^2^/g, respectively, where that of P-CeO_2_-H_2_, C-CeO_2_-H_2_ and R-CeO_2_-H_2_ is 10.41, 16.43, and 44.76 m^2^/g. We found that the BET surface area of all samples decreased after calcining in H_2_, which further indicates that the surface OVs concentration is the major factor on the photocatalytic properties of ceria in this study. In addition, the excellent photocatalytic performance of P-CeO_2_ may be related to the ordered mesoporous structure.

Hence, the similar morphology, size and BET surface area of the annealed ceria in different atmospheres suggests that the surface OVs concentration is the major factor in the photocatalytic properties of ceria in this study.

### 3.5. Band Structure of as Prepared Ceria

**Figure 6:**
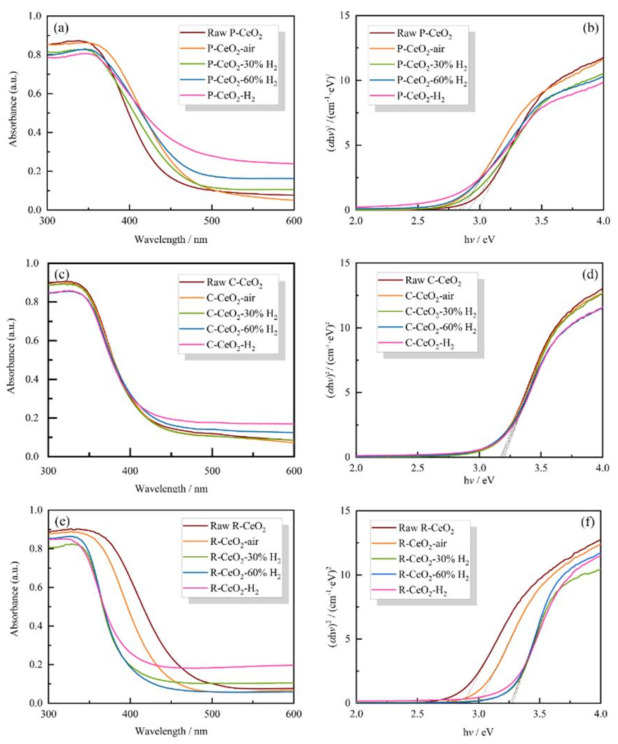
UV-Vis DRS and energy band curves of P-CeO_2_ (**a**,**b**), C-CeO_2_ (**c**,**d**) and R-CeO_2_ (**e**,**f**) calcining in different concentration of H_2_.

The UV-Vis DRS spectra and the band energy curves of CeO_2_ are shown in Figure 6, and the calculated band gap values are given in Table S5, and it is found that the variations of the light absorption behavior and the band gap for the different morphology ceria before and after annealing are not stereotyped. After annealing, the band gap firstly expands and then slightly narrows with the increase of surface OVs concentration. Raw P-CeO_2_ has a band gap of 2.987 eV, while the band gap values of annealed cubic nanoceria are in the range of 2.796–2.864 eV. C-CeO_2_ samples have similar band gaps of 3.170–3.204 eV. Raw R-CeO_2_ has a narrower band gap of 2.882 eV than that of annealed R-CeO_2_ samples, while the band gap value of R-CeO_2_-air increases to 3.019 eV and then turns to 3.283 eV for 30% H_2_ annealed sample, by continually increasing the H_2_ concentration the values tend to decrease slightly. Interestingly, the observed variations of band gap are quite different from the previous reports (e.g., [21,26]), and no significant changes of band gap are observed with the increasing of OVs concentration, which indicates that the surface OVs generated by hydrogen annealing at 600 °C have minimal effects on the band gap of nanoceria.

**Figure 7:**
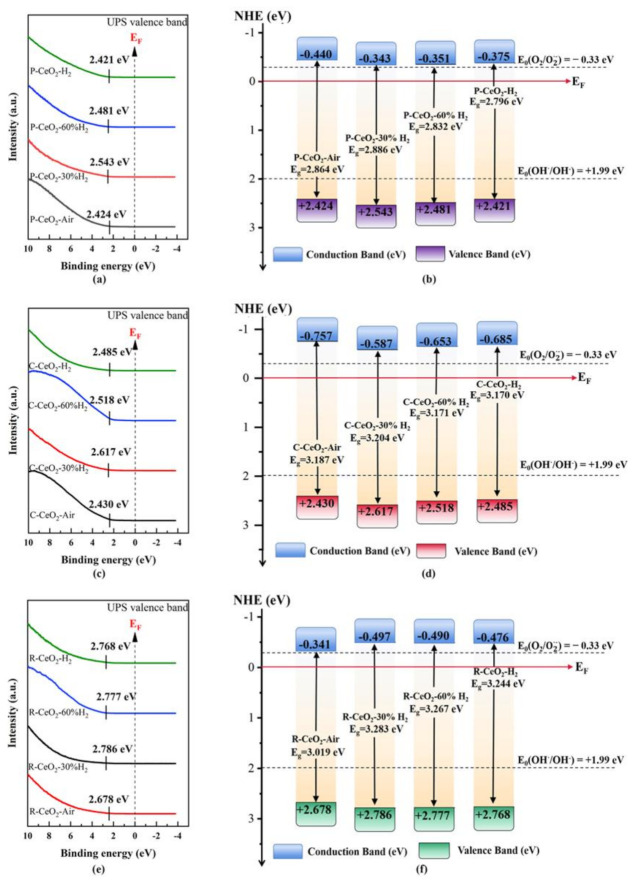
UPS valence band spectra and energy band gap schematic diagram of P-CeO_2_ (**a**,**b**), C-CeO_2_ (**c**,**d**), and R-CeO_2_ (**e**,**f**) annealed in different concentration of H_2_.

Band gap energy (*E_g_*) of ceria depends on the conduction band (CB) and valence band (VB), the energy of VB (*E_VB_*) of annealed ceria was analyzed by UPS and the results are shown in Figure 7, where the band edge position of CB (*E_CB_*) was calculated based on the relationship as given in Equation (5).

*E_VB_* = *E_CB_* + *E_g_*. (5)

Moreover, the value of *E_VB_* and *E_CB_* can be generally calculated by the Mullikan Electronegativity equation [64]:

*E_CB_* = χ − *E_C_* − 1 / 2 *E_g_*, (6)

where χ is the absolute electronegativity of *E_C_* the semiconductor, the χ value of CeO_2_ is reported as 5.56 eV [65], and *E_C_* is the energy of free electrons on the hydrogen scale (−4.5 eV [66]). The measured and calculated *E_VB_* and *E_CB_* are listed in Table S5, where it can be found that the band edge positions obtained under different conditions present a similar variation trend for the same shaped ceria samples. For three studied structured ceria, the *E_VB_* expands to a more positive position when the annealing atmosphere turns to 30% H_2_ from air, then decreases with the rising of H_2_ concentration. On the contrary, the values of *E_CB_* of C-CeO_2_ and P-CeO_2_ are firstly moved to the Fermi level closely and then become more negative with the OVs concentration rising, while the *E_CB_* of R-CeO_2_ is firstly expanded to a more negative position and then turns back to Fermi level with the increase of surface OVs.

### 3.6. Separation/Recombination of e^−^/h^+^

**Figure 8:**
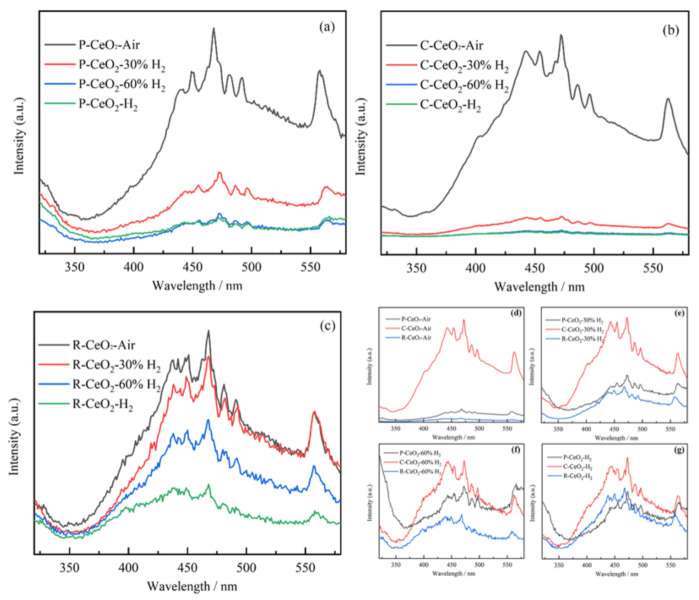
The PL spectra of annealed P-CeO_2_ (**a**), C-CeO_2_ (**b**), R-CeO_2_ (**c**) and the samples annealed in same condition (**d**–**g**).

PL spectra, as shown in Figure 8, were employed to investigate the recombination efficiency of photoinduced electrons and holes, where a lower recombination rate is characterized by a lower PL intensity [17,67]. It can be found that all PL spectra show strong blue emission peaks centered at 430–490 nm, which is associated with the defect levels localized between the Ce 4f and O 2p bands [68,69,70,71]. With the rising of surface OVs concentration, the intensities of PL spectra for P-CeO_2_ and C-CeO_2_ obviously weaken firstly, then decrease slightly. However, the intensities of emission spectra for R-CeO_2_ samples are continually weakening with the increasing of OVs concentration, which may offer an evidence for the potential or further reducing of the recombination rate of photogenerated carrier for ceria nanorod with a higher surface OVs concentration. Besides, after annealing in air, 30% and 60% H_2_, R-CeO_2_ shows a lower PL intensity than that of other typical structure nanoceria, while after annealing in pure hydrogen, the P-CeO_2_ exhibits the lowest PL intensity.

**Figure 9:**
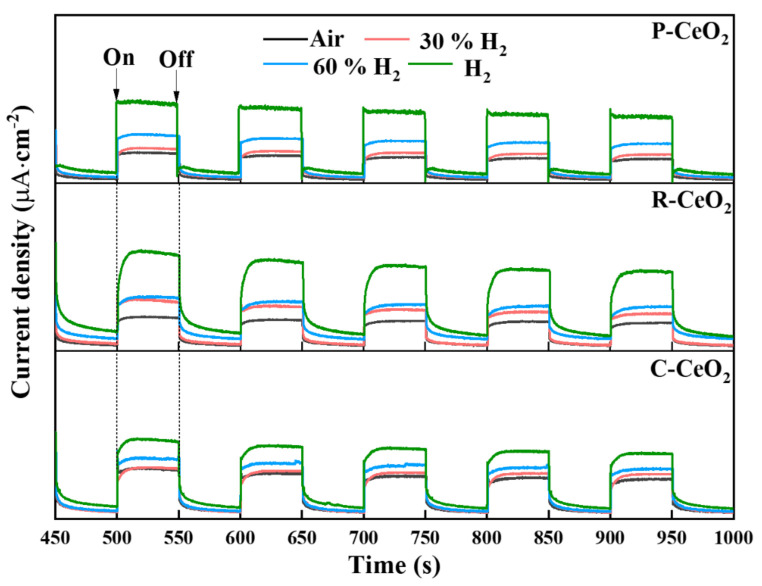
The transient photocurrent curves of P-CeO_2_, C-CeO_2_ and R-CeO_2_ calcining in different concentration of H_2_.

In order to further confirm the separation efficiency of photogenerated electron-hole pairs of the studied samples [72], the transient photocurrent response experiments were measured, and the average photocurrent densities are shown in Figure 9 and Table S6. Higher photocurrent densities are presented with the rising of surface OVs concentration in P-CeO_2_, C-CeO_2_ and R-CeO_2_ annealed in increasing concentration of H_2_, which suggests a higher surface OVs concentration may elevate the e^−^/h^+^ separation efficiency of CeO_2_ photocatalyst. It is generally known that a higher separation and lower recombination rate of e^−^/h^+^ are beneficial to the better photocatalytic activity [73], which provide further evidence for the enhancement of photocatalytic activity of ceria after annealing in hydrogen.

### 3.7. Proposed Mechanism for Photocatalytic Enhancement

To evaluate the contributions of surface OVs on the photoelectric characterizations and photocatalytic activity of ceria, the offset values of each property of different hydrogen annealed ceria compared with the air annealed CeO_2_ were calculated using the following equation:

*Offset Value* = (*P*_*i*_ − *P*_0_)/*P*_0_ × 100%, (7)

where *P* means the properties including band gap value, photodegradation ratio of MB at 2 h or the photogenerated current, *i* (*i* = 0, 1, 2, and 3) represents the number of annealed samples, where 0, 1, 2, and 3 means the sample annealed in air, 30%, 60%, and pure H_2_, respectively.

**Figure 10:**
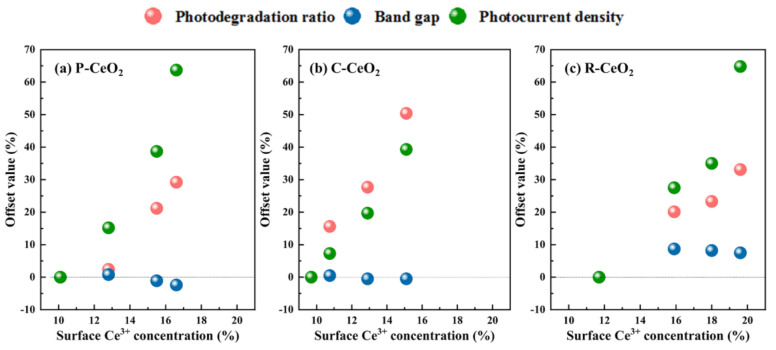
The contributions of Ce^3+^ on the photoelectric characterizations and photocatalytic activity.

The relationships of offset values vs. surface Ce^3+^ concentration of ceria are shown in Figure 10 and Table S7. The offset value of band gap is slightly decreasing with the increasing of surface Ce^3+^ while the 30% H_2_ annealed samples have a wider band gap than that of air annealed ceria, suggesting that surface OVs may expand the band gap of ceria firstly, and then the band gap value tends to decrease slightly with a continual rising of surface OVs. Revealed results may explain the reported references, e.g., Gao et al. [22] obtained rich surface OVs ceria by surface engineering with a blue shift of the UV-Vis spectra. Interestingly, the variations both of the offset value of photodegradation ratio and the photocurrent density are notably rising with an increase of surface OVs, which indicates that the reduction of e^−^/h^+^ recombination may be the major contribution of surface OVs on the enhancement of photocatalytic activity under same light source.

Comparing the effects of surface OVs on different shaped ceria, it can be found that surface OVs affect the photocatalytic activity most significantly on the cubic ceria, while C-CeO_2_ contains low surface OVs due to its large particle size and high activity of (100) facet, more surface OVs induced in ceria nanocube lattice may result in a moderate photocatalytic activity. Even the effect of surface OVs working on photocatalytic activity of R-CeO_2_ is slightly smaller than that of P-CeO_2_, but more OVs can be generated in the R-CeO_2_, which also results in a high photocatalytic activity. On the other hand, the polyhedral ceria has the smallest size distribution, which may be one of important reasons for its excellent photocatalytic property.

**Figure 11:**
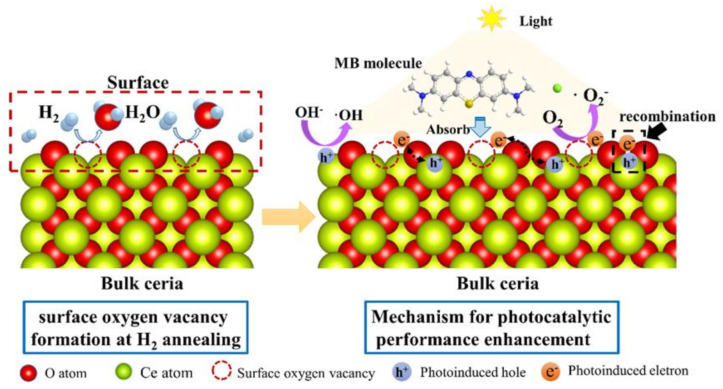
Mechanism schematic of promoted photocatalytic activity of surface contained nanoceria.

Based on the revealed results, the contributions of surface OVs on the photocatalytic activity of ceria can be concluded as shown in Figure 11. In the range of studied surface OV concentration in cubic, polyhedral or rod-like ceria, a significantly reduction of the combination of e^−^/h^+^ is the major contribution of surface OVs on the promoted photocatalytic activity, while the band gap varies slightly. The surface OVs in CeO_2_ lattice are rearranged to produce small microdomains [74] and ordered together to form electron deep traps which can facilitate the reduction of the recombination rate between photoelectrons and holes during the photocatalytic process [24,75]. Moreover, surface OVs profit the adsorption of O_2_ or OH^−^ on ceria surface, which will promote the generation of radical and reduce the recombination of e^−^/h^+^ photocarriers [69]. Hence, under the same illumination condition, the photocatalytic activity is obviously enhanced with the rising of surface OVs concentration, which is majorly influenced by the reduced recombination of e^−^/h^+^. In addition, the effect rule of surface OVs on photoelectric characterizations and photocatalytic activity of cubic, polyhedral and rod-like ceria is similar but with different incidence, furthermore, reducing particle size and gaining OVs concentration of ceria are still the major tactics for enhancing its photocatalytic activity.

## 4. Conclusions

After sufficient discussion of the revealed results, it can be concluded that a concentration gradient of surface OVs can be generated in ceria lattice after annealing nanoceria at 600 °C in various H_2_ concentration atmospheres, and the ceria annealed in hydrogen has a larger particle size and the exposing lattice face tuned after annealing. Surface OVs significantly enhanced the photocatalytic activity of ceria, the MB degradation ratio after 2 h with pure hydrogen annealed C-CeO_2_, P-CeO_2_, or R-CeO_2_ is 85.15%, 93.82% and 90.09%, respectively, which is 1.5, 1.29 and 1.33 times higher than that of the air annealed sample. The band structure, including band gap, VB, and CB of annealed samples vary slightly, even the surface OVs in ceria lattice changed obviously. Recombination of photoinduced carrier, e^−^/h^+^, has a notable reduction with the rising of surface OVs, which is suggested to be the main contribution for the enhancement of photocatalytic activity of ceria with more surface OVs.

## Data Availability

Data is contained within the article and supplementary material.

## Supplementary Materials

The following are available online at https://www.mdpi.com/article/10.3390/nano11051168/s1, Figure S1. Size distribution of raw P-CeO_2_ (**a**), C-CeO_2_ (**b**) and R-CeO_2_ (**c**). Figure S2. Color variation of P-CeO_2_, C-CeO_2_ and R-CeO_2_ (from left to right) calcining in different atmospheres: (**a**) raw samples without annealing, (**b**) air, (**c**) 30% H_2_, (**d**) 60% H_2_, and (**e**) pure H_2_ condition. Figure S3. O 1s spectra of P-CeO_2_ (a), C-CeO_2_ (**b**) and R-CeO_2_ (**c**) calcining in different concentration of H_2_. Figure S4. TEM images and size distribution of P-CeO_2_ calcining in air (**a**–**c**) and H_2_ (**d**–**f**) at 600 °C. Figure S5. TEM images and size distribution of C-CeO_2_ calcining in air (**a**–**c**) and H_2_ (**d**–**f**) at 600 °C. Figure S6. TEM images and size distribution of R-CeO_2_ calcining in air (**a**–**c**) and H_2_ (**d**–**f**) at 600 °C. Figure S7. TEM images of raw R-CeO_2_ (**a**), R-CeO_2_-Air (**b**) and R-CeO_2_-H_2_ (**c**). Figure S8. The XRD patterns of C-CeO_2_ annealed in air, 30%, 60% and pure H_2_. Figure S9. Nitrogen adsorption–desorption isotherms of the P-CeO_2_ (**a**), C-CeO_2_ (**b**), and R-CeO_2_ (**c**) calcining in air or pure H_2_. Table S1. Synthetic conditions of raw P-CeO_2_, C-CeO_2_ and R-CeO_2_. Table S2. Ce^3+^ and absorbed oxygen concentration of P-CeO_2_, C-CeO_2_ and R-CeO_2_ calcining in different concentration of H_2_. Table S3. Photocatalytic degradation ratio and rate constants of P-CeO_2_, C-CeO_2_, and R-CeO_2_ calcining in different atmospheres. Table S4. The calculated crystal size of C-CeO_2_ annealed in air, 30%, 60% and pure H_2_. Table S5. Energy band gap, calculated valence and conductive band, tested valence and conductive band of P-CeO_2_, C-CeO_2_, and R-CeO_2_ calcining in different concentration of H_2_. Table S6. Average current density (μA/cm^2^) of P-CeO_2_, C-CeO_2_, and R-CeO_2_ calcining in different concentration of H_2_. Table S7. Offset values of photodegradation ratio, band gap and photocurrent density of different hydrogen annealed ceria.
