# Supplementary material for: Insight into the Contributions of Surface Oxygen Vacancies on the Promoted Photocatalytic Property of Nanoceria
Source: Nanomaterials (Basel). 2021 Apr 29;11(5):1168. doi: 10.3390/nano11051168 (PMC8145243; doi:10.3390/nano11051168)
Supplement: Supplementary file 1 [file nanomaterials-11-01168-s001.zip › nanomaterials-1180013 SI updated.pdf]

nanomaterials

Supplementary Information

Insight into the Contributions of Surface Oxygen Vacancies on the Promoted Photocatalytic Property of Nanoceria

Yuanpei Lan [1,2], Xuewen Xia [1,2], Junqi Li [1,2,*], Xisong Mao [1,2], Chaoyi Chen [1,2,*], Deyang Ning [1,2], Zhiyao Chu [1,2], Junshan Zhang [1,2] and Fengyuan Liu [1,2]

1 Department of Metallurgical Engineering, College of Materials and Metallurgy, Guizhou University, Huaxi, Guiyang, Guizhou 550025, China; yplan@gzu.edu.cn (Y.L.); XuewenXiaCN@outlook.com (X.X.); gz-maoxisong@outlook.com (X.M.); ndy1113@gmail.com (D.N.); ZhiyaoChuCN@outlook.com (Z.C.); JunshanZhangCN@outlook.com (J.Z.); FengyuanLiu-CN@outlook.com (F.L.)
2 Guizhou Province Key Laboratory of Metallurgical Engineering and Process Energy Saving, Guiyang, Guizhou 550025, China
* Correspondence: ccy197715@126.com (C.C.); jqli@gzu.edu.cn (J.L.); Tel.: +86-15086015817 (C.C.); Tel.: +86-13594152275 (J.L.)

The synthetic conditions for morphology turning of ceria are listed in **Table S1**, the polyhedral, cubic and rod-like shape of nanoceria can be obtained by adjusting the concentration of NaOH solution and synthetic temperature according to the Ref. [1].

Table S1. Synthetic conditions of raw P-CeO_2, C-CeO_2 and R-CeO_2.

NO.	m($Ce(NO_3)_3·6H_2O$) / g	m(NaOH) / g	Time / h	Temperature / °C
P-CeO_2	0.868	0.160	24	100
C-CeO_2	0.868	9.600	24	180
R-CeO_2	0.868	9.600	24	100

The particle sizes of synthesized ceria are counted based on the TEM images and around 80 particles were employed for statistical analyzing, and the results are given in **Figure S1**, where it can be found that the size of raw P-CeO_2, C-CeO_2 and R-CeO_2 is around 9, 40, and 100 nm (for length) respectively.

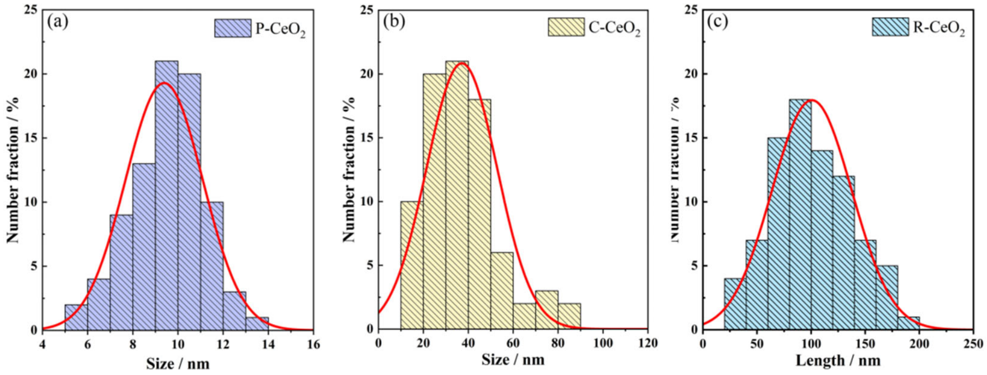

Figure S1. Size distribution of raw P-CeO_2 (a), C-CeO_2 (b) and R-CeO_2 (c).

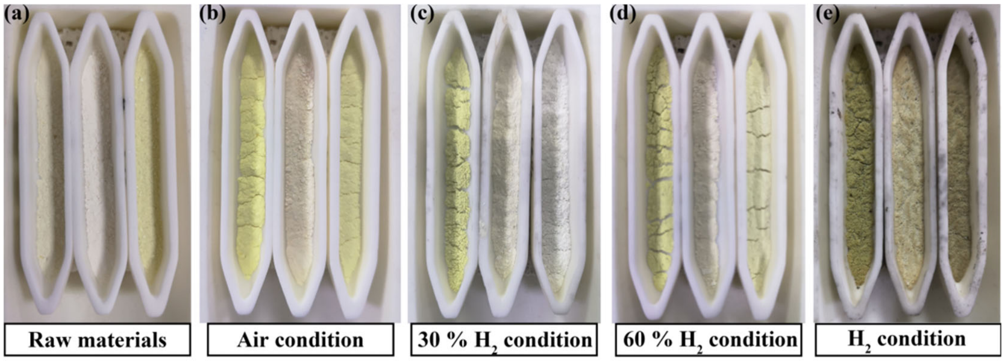

Figure S2. Color variation of P-CeO_2, C-CeO_2 and R-CeO_2 (from left to right) calcining in different atmosphere: (**a**) raw samples without annealing, (**b**) air, (**c**) 30 % H_2, (**d**) 60 % H_2 and (**e**) pure H_2 condition.

Obvious color changes can be observed after annealing, and the ceria annealed in air is pale yellow, and then turn to yellow-green, glaucous and blue-yellow after annealing in 30 %, 60 % and pure hydrogen, and the colors are shown in **Figure S2**. It is known that stoichiometric cerium dioxide is pale yellow [2], and the color will turn to blue or even black after the formation of nonstoichiometric ceria [3], the observed color change means the OVs are generated after hydrogen annealing.

The XPS spectra and deconvoluted O 1s results are shown in **Figure S3**. It can be seen that all samples own similar XPS spectra but distinguishable difference in O 1s curves. Two different types of oxygen species assign to the lattice oxygen (O_L) and the oxygen ions adsorbed on the surface (O_V) [4], The area and intensity of O_V peak is relevant to the oxygen vacancy in the host lattice. The O_V peak is slightly increasing while the O_L peak is somewhat decreasing with the increasing of H_2 concentration in annealing atmosphere. The fractions of O_V have been calculated by following equation:

$$\frac{0_V}{(0_V + 0_L)} = \frac{\text{area}(0_V)}{\text{total area}} \tag{1}$$

The results are listed in **Table S2** together with the Ce^{3+} fractions.

It can be found that the Ce^{3+} and absorbed oxygen (O_V fraction) concentrations of P-CeO_2, C-CeO_2 and R-CeO_2 are increased with the rising of hydrogen concentration in annealing atmosphere.

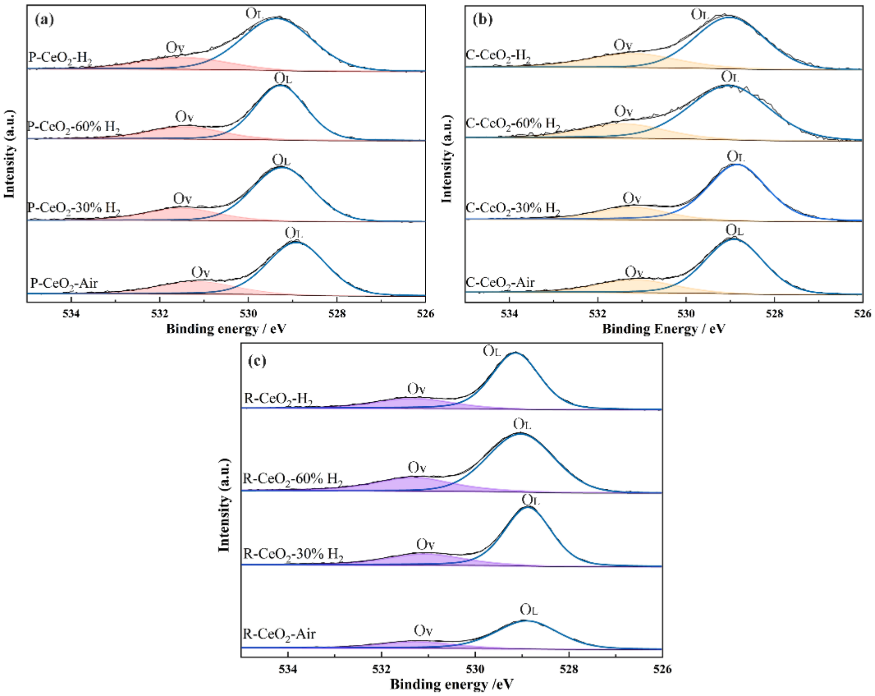

Figure S3. O 1s spectra of P-CeO_2 (**a**), C-CeO_2 (**b**) and R-CeO_2 (**c**) calcining in different concentration of H_2. **Table S2.** Ce^{3+} and absorbed oxygen concentrations of P-CeO_2, C-CeO_2 and R-CeO_2 calcining in different concentration of H_2.

No.	Air		30 % H_2		60 % H_2		H_2	
	Ce^{3+}, %	O_V, %	Ce^{3+}, %	O_V, %	Ce^{3+}, %	O_V, %	Ce^{3+}, %	O_V, %
P-CeO_2	10.1 %	21.7 %	12.8 %	23.5 %	15.5 %	25.2 %	16.6 %	26.6 %
C-CeO_2	9.7 %	13.7 %	10.7 %	20.8 %	12.9 %	21.3 %	15.1 %	23.6 %
R-CeO_2	11.7 %	21.5 %	15.9 %	21.8 %	18.0 %	22.2 %	19.6 %	22.9 %

Table S3. Photocatalytic degradation ratio and rate constants of P-CeO_2, C-CeO_2, and R-CeO_2 calcining in different atmosphere.

NO.	C/C_0	k / min^{-1}
Raw P-CeO_2	51.29 %	0.00484
P-CeO_2-Air	72.60 %	0.00844
P-CeO_2-30 % H_2	74.37 %	0.00912
P-CeO_2-60 % H_2	88.02 %	0.01386
P-CeO_2-H_2	93.82 %	0.01787
Raw C-CeO_2	42.18 %	0.00373
C-CeO_2-Air	56.63 %	0.00568
C-CeO_2-30 % H_2	65.47 %	0.00694
C-CeO_2-60 % H_2	72.31 %	0.00886
C-CeO_2-H_2	85.15 %	0.01229
Raw R-CeO_2	61.16 %	0.00652
R-CeO_2-Air	67.69 %	0.00796
R-CeO_2-30% H_2	81.32 %	0.01125
R-CeO_2-60% H_2	83.45 %	0.01172
R-CeO_2-H_2	90.09 %	0.01572

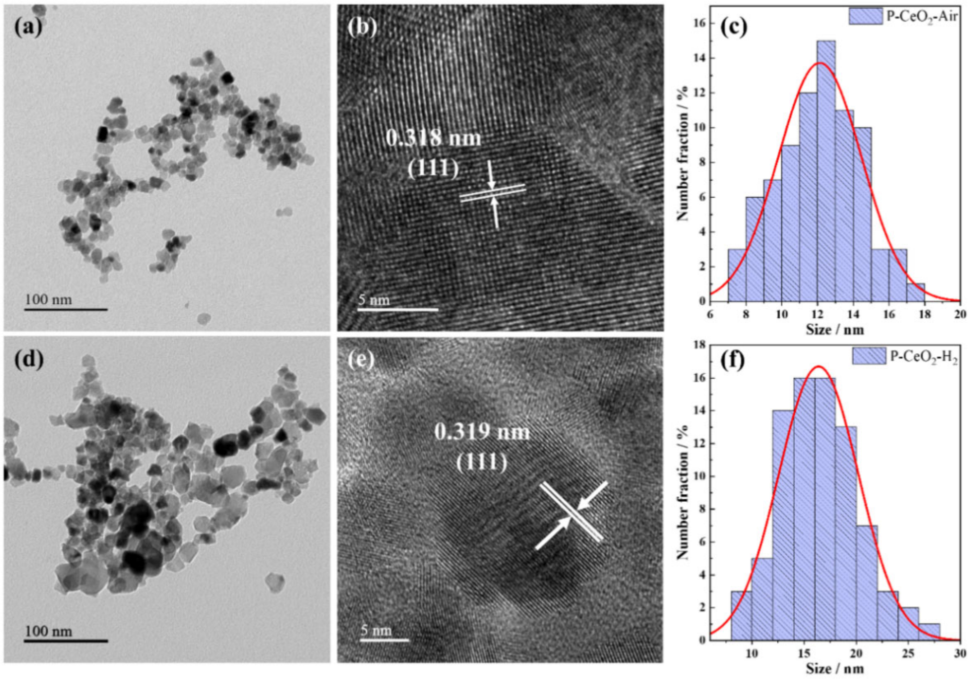

Figure S4. TEM images and size distribution of P-CeO_2 calcining in air (**a**–**c**) and H_2 (**d**–**f**) at 600 °C.

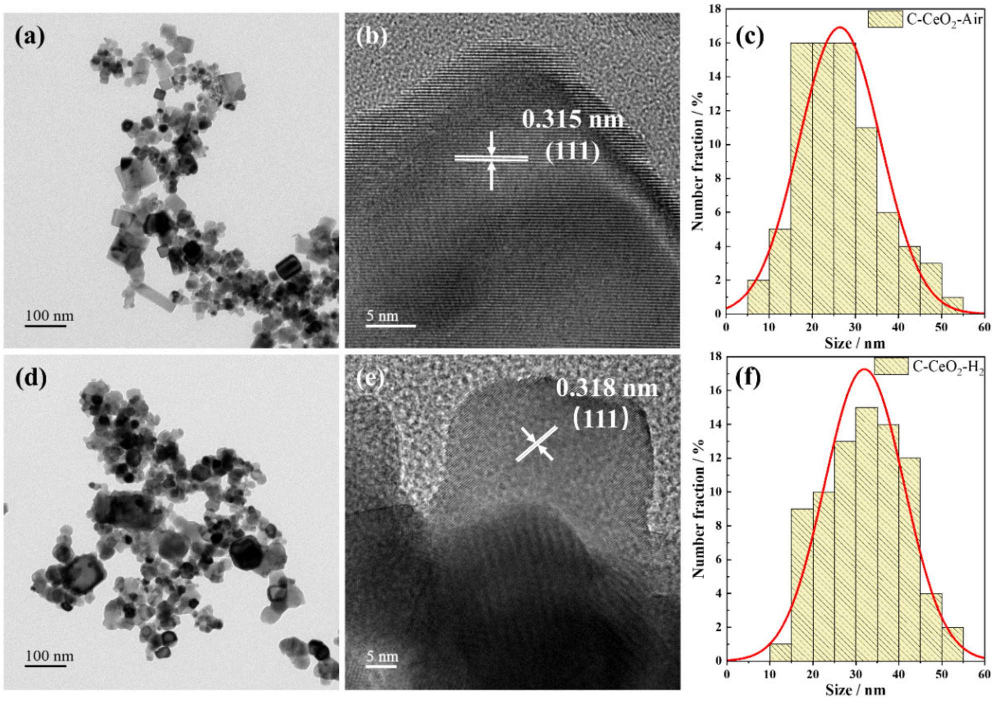

Figure S5. TEM images and size distribution of C-CeO_2 calcining in air (**a**–**c**) and H_2 (**d**–**f**) at 600 °C.

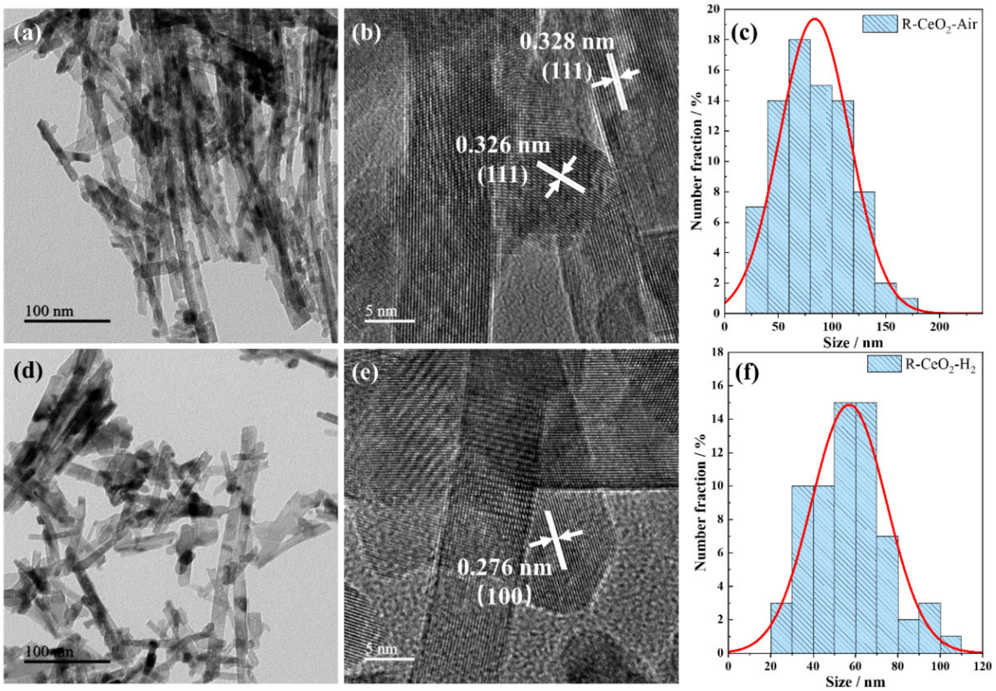

Figure S6 TEM images and size distribution of R-CeO_2 calcining in air (**a**–**c**) and H_2 (**d**–**f**) at 600 °C

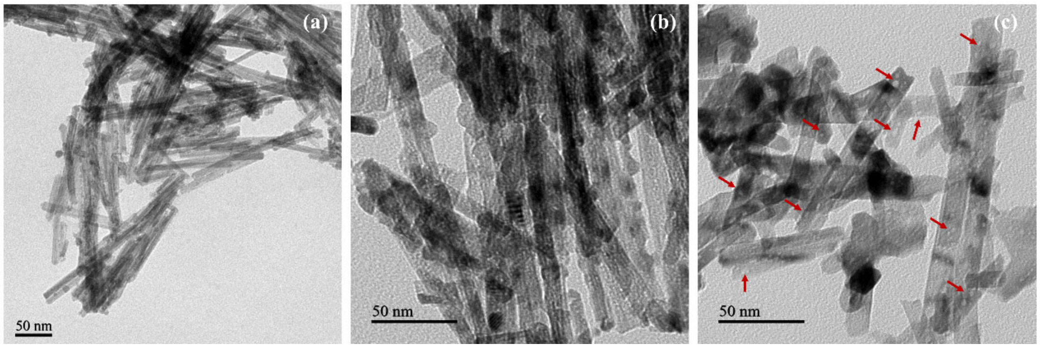

Figure S7. TEM images of raw R-CeO_2 (**a**), R-CeO_2-Air (**b**) and R-CeO_2-H_2 (**c**).

The XRD patterns of C-CeO_2 annealed in air, 30 %, 60 % and pure H_2 have been added and the results are shown in **Figure S8**. It can be found that all annealed samples have the similar diffraction pattern of CeO_2, no peaks of Ce_2O_3 can be found. The calculated crystal size is given in **Table S4**

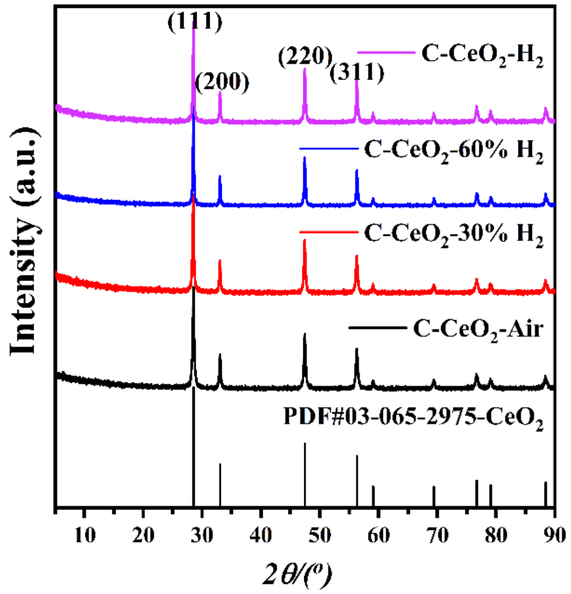

Figure S8. The XRD patterns of C-CeO_2 annealed in air, 30 %, 60% and pure H_2.

Table S4. The calculated crystal size of C-CeO_2 annealed in air, 30 %, 60 % and pure H_2.

NO.	C-CeO2-Air	C-CeO2-30 %H2	C-CeO2- 60 %H2	C-CeO2-H2
Crystal Size/ (nm)	31.7	36.1	52.9	47.3

The nitrogen adsorption–desorption isotherms of the P-CeO_2 (a), C-CeO_2 (b), and R-CeO_2 (c) calcining in air or pure H_2 are shown in Figure S9. It was calculated that the BET surface area of P-CeO_2-Air, C-CeO_2-Air and R-CeO_2-Air is 60.25, 20.94 and 68.20 m^2/g respectively, where that of P-CeO_2-H_2, C-CeO_2-H_2 and R-CeO_2-H_2 is 10.41, 16.43, and 44.76 m^2/g.

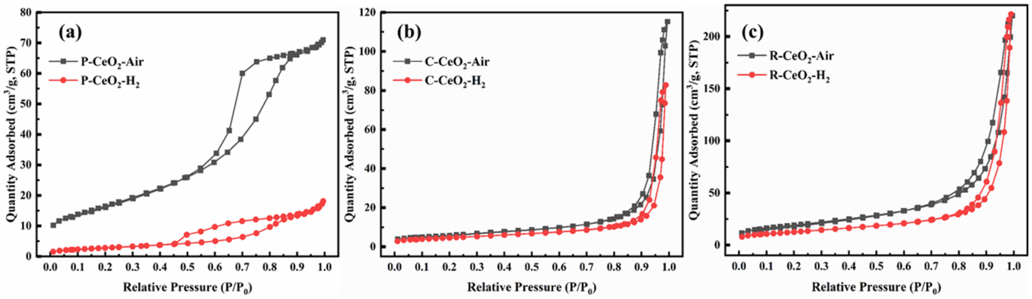

Figure S9. Nitrogen adsorption–desorption isotherms of the P-CeO_2 (**a**), C-CeO_2 (**b**), and R-CeO_2 (**c**) calcining in air or pure H_2.

The measured E_{VB} and E_{CB} by UPS and UV-DRS, as the calculated values by Mullikan Electronegativity equation [5] are given in **Table S5**. It can be found that the band edge positions obtained by different methods present similar variation trends for same shaped ceria samples.

Table S5. Energy band gap, calculated valence and conductive band, tested valence and conductive band of P-CeO_2, C-CeO_2, and R-CeO_2 calcining in different concentration of H_2.

NO.	Energy band gap (eV)	Calculated valence band (eV)	Calculated conductive band (eV)	valence band (eV)	conductive band (eV)
Raw P-CeO_2	2.987	2.554	-0.434	-	-
P-CeO_2-Air	2.864	2.492	-0.372	2.424	-0.440
P-CeO_2-30 % H_2	2.886	2.503	-0.383	2.543	-0.343
P-CeO_2-60 % H_2	2.832	2.476	-0.356	2.481	-0.351
P-CeO_2-H_2	2.796	2.458	-0.338	2.421	-0.375
Raw C-CeO_2	3.170	2.645	-0.525	-	-
C-CeO_2-Air	3.187	2.654	-0.535	2.430	-0.757
C-CeO_2-30 % H_2	3.204	2.662	-0.542	2.617	-0.587
C-CeO_2-60 % H_2	3.171	2.646	-0.526	2.518	-0.653
C-CeO_2-H_2	3.170	2.645	-0.525	2.485	-0.685
Raw R-CeO_2	2.882	2.501	-0.381	-	-
R-CeO_2-Air	3.019	2.570	-0.450	2.678	-0.341
R-CeO_2-30 % H_2	3.283	2.702	-0.582	2.786	-0.497
R-CeO_2-60 % H_2	3.267	2.694	-0.574	2.777	-0.490
R-CeO_2-H_2	3.244	2.682	-0.562	2.768	-0.476

Table S6. Average current density ($\mu A/cm^2$) of P-CeO_2, C-CeO_2, and R-CeO_2 calcining in different concentration of H_2.

No.	Air	30 % H_2	60 % H_2	H_2
P-CeO_2	0.613	0.723	1.000	1.690
C-CeO_2	1.020	1.100	1.270	1.680
R-CeO_2	0.715	0.986	1.100	2.030

Table S7. Offset values of photodegradation ratio, band gap and photocurrent density of different hydrogen annealed ceria.

NO.	Surface Ce^{3+} concentration	Photodegradation ratio	Band gap	Photocurrent density
P-CeO_2-Air	10.1 %	0 %	0 %	0 %
P-CeO_2-30 % H_2	12.8 %	2.4 %	0.8 %	15.2 %
P-CeO_2-60 % H_2	15.5 %	21.2 %	-1.1 %	38.7 %
P-CeO_2-H_2	16.6 %	29.2 %	-2.4 %	63.7 %
C-CeO_2-Air	9.7 %	0 %	0 %	0 %
C-CeO_2-30 % H_2	10.7 %	15.6. %	0.5 %	7.3 %
C-CeO_2-60 % H_2	12.9 %	27.7 %	-0.5 %	19.7 %
C-CeO_2-H_2	15.1 %	50.4 %	-0.5 %	39.3 %
R-CeO_2-Air	11.7 %	0 %	0 %	0 %
R-CeO_2-30% H_2	15.9 %	20.1 %	8.7 %	27.5 %

R-CeO_2-60% H_2	18.0 %	23.1 %	8.2 %	35.0 %
R-CeO_2-H_2	19.6 %	33.1 %	7.5 %	64.8 %

References

1. Mai, H.X.; Sun, L.D.; Zhang, Y.W.; Si, R.; Feng, W.; Zhang, H.P.; Liu, H.C.; Yan, C.H. Shape-Selective Synthesis and Oxygen Storage Behavior of Ceria Nanopolyhedra, Nanorods, and Nanocubes. *Journal of Physical Chemistry B* **2005**, *109*, 24380–24385.
2. Neish, A.C. PREPARATION OF PURE CERIUM SALTS AND THE COLOR OF CERIUM OXIDE. 1. *J Am Chem Soc* **1909**, *31*, 517–523.
3. Mogensen, M.; Sammes, N.M.; Tompsett, G.A. Physical, chemical and electrochemical properties of pure and doped ceria. *Solid State Ionics* **2000**, *129*, 63–94.
4. Fan, L.; Wang, K.; Xu, K.; Liang, Z.; Wang, H.; Zhou, S.F.; Zhan, G. Structural Isomerism of Two Ce-BTC for Fabricating Pt/CeO_2 Nanorods toward Low-Temperature CO Oxidation. *Small* **2020**, *16*, 2003597.
5. Islam, N., Jahurul; D., A.R.; Choi, J.; Kim, T. Surface oxygen vacancy assisted electron transfer and shuttling for enhanced photocatalytic activity of a Z-scheme CeO_2–AgI nanocomposite. *Rsc Advances* **2016**, *6*, 19341–19350.
